# The landscape of *SETBP1* gene expression and transcription factor activity across human tissues

**DOI:** 10.1371/journal.pone.0296328

**Published:** 2024-01-02

**Authors:** Jordan H. Whitlock, Elizabeth J. Wilk, Timothy C. Howton, Amanda D. Clark, Brittany N. Lasseigne

**Affiliations:** Department of Cell, Developmental and Integrative Biology, Heersink School of Medicine The University of Alabama at Birmingham, Birmingham, Alabama, United States of America; Free University of Berlin, GERMANY

## Abstract

The SET binding protein 1 (*SETBP1*) gene encodes a transcription factor (TF) involved in various cellular processes. Variants in *SETBP1* can result in three different diseases determined by the introduction (germline vs. somatic) and location of the variant. Germline variants cause the ultra-rare pediatric Schinzel Giedion Syndrome (SGS) and *SETBP1* haploinsufficiency disorder (*SETBP1*-HD), characterized by severe multisystemic abnormalities with neurodegeneration or a less severe brain phenotype accompanied by hypotonia and strabismus, respectively. Somatic variants in *SETBP1* are associated with hematological malignancies and cancer development in other tissues in adults. To better understand the tissue-specific mechanisms involving *SETBP1*, we analyzed publicly available RNA-sequencing (RNA-seq) data from the Genotype-Tissue Expression (GTEx) project. We found *SETBP1* and its known target genes were widely expressed across 31 adult human tissues. K-means clustering identified three distinct expression patterns of SETBP1 targets across tissues. Functional enrichment analysis (FEA) of each cluster revealed gene sets related to transcriptional regulation, DNA binding, and mitochondrial function. TF activity analysis of SETBP1 and its target TFs revealed tissue-specific TF activity, underscoring the role of tissue context-driven regulation and suggesting its impact in SETBP1-associated disease. In addition to uncovering tissue-specific molecular signatures of *SETBP1* expression and TF activity, we provide a Shiny web application to facilitate exploring TF activity across human tissues for 758 TFs. This study provides insight into the landscape of *SETBP1* expression and TF activity across 31 non-diseased human tissues and reveals tissue-specific expression and activity of *SETBP1* and its targets. In conjunction with the web application we constructed, our framework enables researchers to generate hypotheses related to the role tissue backgrounds play with respect to gene expression and TF activity in different disease contexts.

## Introduction

*SETBP1* is a gene located on the long (q) arm of chromosome 18 that encodes the transcription factor (TF) and oncogene SET binding protein 1 [1]. Referred to as a DNA-binding protein, SETBP1 has several motifs, including three nuclear localization signals, a SKI homology region, and a binding region for SET nuclear oncogene. As a protein, it has a role in DNA replication and transcriptional regulation [2]. SETBP1 binds the SET nuclear oncogene whose SET binding domain is involved in DNA replication [3]. Furthermore, the AT hooks of SETBP1 allow DNA binding and gene expression activation through the formation of an epigenetic complex composed of SETBP1, PHF8, KMT2A, and HCF1 [2]. Different pathogenic variants in *SETBP1* can result in three distinct diseases [4]. Germline variants cause two unique ultra-rare, de novo pediatric diseases: Schinzel Giedion Syndrome (SGS) [5] and SETBP1 haploinsufficiency disorder (*SETBP1*-HD) [6]. These conditions are differentiated by variant location, phenotypic severity, and accompanying protein gain or loss of function (GoF, LoF), respectively [5, 6]. SGS is multisystemic, involving gastrointestinal, cardiorespiratory, neurological, musculoskeletal, and urogenital abnormalities. It has a more severe phenotype than *SETBP1*-HD, and affected individuals are characterized by progressive neurodegeneration and shortened life expectancy [7, 8]. However, SGS and *SETBP1*-HD, as disorders of protein dosage, have overlapping phenotypes, including intellectual disability, developmental delay, language impairment, distinctive craniofacial and skeletal features, and hypotonia [5–7]. In contrast, somatic variants in *SETBP1* are associated with hematological malignancies and exhibit varying evidence for predisposing or promoting cancer in other adult tissue systems (reviewed in [1]).

There are multiple hypothesized mechanisms for the tissue-specificity of disease (i.e., clinical manifestations in some tissues but not others) related to intrinsic and extrinsic molecular processes spanning epigenetic, genetic, expression, regulation, and network-based mechanisms [9]. Despite genomic advances in variant identification and sequencing technology, gaps remain in translating the role of specific genomic variants to observed phenotypic outcomes. Of these hypothesized mechanisms of tissue-specific disease manifestation, preferential or exclusive gene expression of SETBP1 targets and their altered regulation remain understudied in SETBP1-associated disorders. Potential mechanisms of SETBP1 dysfunction within neurodevelopment involving disrupted cell cycle control, DNA damage mechanisms, phosphatase activity, and chromatin remodeling have been hypothesized and further studied in human stem cells, peripheral blood leukocytes, cell lines, and animal models [10–14]. However, how the expression of *SETBP1* and its known TF targets function across additional tissue contexts and non-diseased human tissues requires further study.

**Fig 1:**
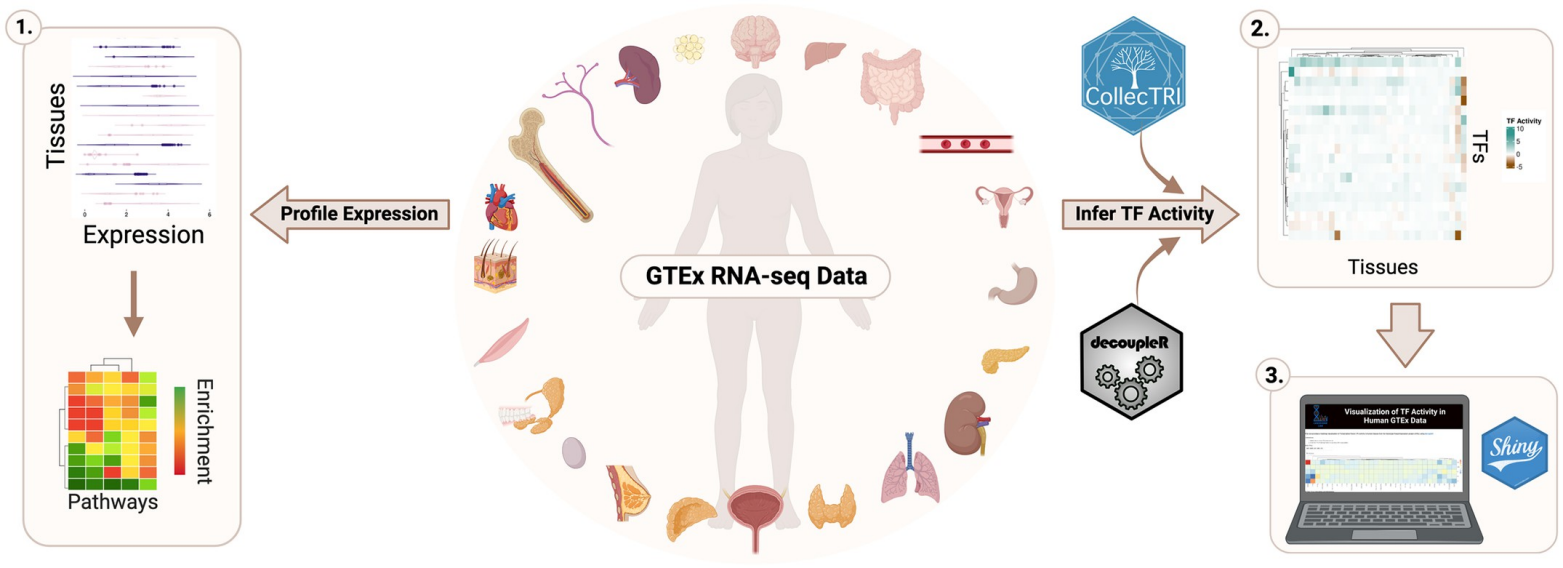
Study overview. To investigate the tissue-specific expression and TF activity of *SETBP1*, we analyzed publicly available RNA-seq data from the GTEx project. We profiled the tissue-specific expression of *SETBP1* and its known targets and performed functional enrichment analysis (1). We also inferred TF activity for SETBP1 and TFs it regulates by tissue (2) and developed an interactive web application to enable the exploration of TF activity for 758 TFs across all GTEx tissues (3).

Because of this, publicly available non-diseased data, such as from the Genotype-Tissue Expression (GTEx) project, provide an opportunity for investigating and generating hypotheses about the underlying function of disease-associated genes in different contexts, including in non-diseased and diseased contexts. Here, we investigated the gene expression of *SETBP1* and its known targets, previously compiled in Whitlock et al. 2023, in RNA-sequencing (RNA-seq) data for each GTEx tissue, with tissues annotated as being affected or unaffected in a SETBP1-associated disease as described in OMIM and previously published literature [5, 6, 8, 15–19]. Then, we evaluated the functional enrichment of those targets based on how they clustered by expression across tissues. Next, we inferred the TF activity of SETBP1 and other TFs it is known to directly target by leveraging multivariate linear models to calculate enrichment scores representing activity for all TFs across tissues using GTEx expression and CollecTRI, a curated collection of TFs and their directional regulation on transcriptional targets (Fig 1). Collectively, we have mapped the gene expression and activity of SETBP1 and its targets across human non-diseased tissues, underscoring the potential impact of tissue background. Further, we have developed a Shiny web application (https://lasseignelab.shinyapps.io/gtex_tf_activity/) to facilitate the exploration and hypothesis generation of TF activity across human tissues for 758 TFs **(**S1 Table).

## Materials & methods

### SETBP1 target gene set construction

We used the SETBP1 target gene set compiled in Whitlock et al. 2023 to obtain a list of known TF targets of SETBP1 [11]. We converted a list of human HGNC to human Ensembl IDs using gprofiler2 [20] (v.0.2.1), resulting in a final list of 209 genes.

### RNA-sequencing expression data

Using recount3 [21] (accessed August 2022), we obtained bulk RNA-seq data represented as transcripts per million (TPM) counts for human tissues (n = 31) from the publicly available GTEx project. We included all tissues not classified as “study_na”.

### Classification of disease-associated affected tissues

We compiled a list of affected tissues in SGS, SETBP1-HD, and SETBP1-associated cancer based on the literature, clinical manifestations noted within Online Mendelian Inheritance of Man (OMIM) (MIM #: 616078, 269150), and UpToDate [5, 6, 8, 15–19]. Affected tissues for each SETBP1-associated disease phenotype included the following:

- SETBP1-HD: brain and muscle
- SGS: brain, muscle, heart, kidney, bladder, lung, small intestine, stomach, esophagus
- SETBP1-associated cancer: bone marrow and blood

While individuals with SGS and SETBP1-HD also present with vision problems and distinctive craniofacial and skeletal abnormalities, ocular and additional bone tissues are not present within GTEx v8 so they were not included in our analyses.

### SETBP1 and target gene expression and pathway enrichment

We calculated the median TPM of *SETBP1* and its targets across samples in each tissue from GTEx and scaled (log2 + 1 transformed) for visualizations. We performed complete linkage of Euclidean distances for hierarchical clustering of GTEx tissues. For target gene expression, we identified optimal k-means clusters by plotting the total within-cluster sum of squared distances between samples (inertia) for each cluster tested (k = 1–15). We identified an elbow at 3 clusters, indicating a decrease in inertia and a sufficient trade-off between information and the number of clusters captured based on the expression of *SETBP1* and targets and plotted median scaled TPM values (S1 Fig). We further verified 3 clusters to be sufficient using the Trace(W) method, which uses the trace (sum of the diagonal) of the dispersion matrix (W), finds the second differences, and selects the cluster with the maximum value between indices (S1 Fig) [22, 23]. To visualize this clustering and the expression of SETBP1 and its targets across GTEx tissues, we used ComplexHeatmap (version 2.10.0) [24]. We next performed functional enrichment analysis (FEA) with gprofiler2 (version 0.2.1) [20, 25] with GO sources (GO:BP, GO:MF, and GO:CC) to identify the expression of pathways from *SETBP1* target genes for each cluster. We applied the Bonferroni procedure for multiple hypothesis correction and used a p-adjusted threshold of 0.05 and, for the background gene list, included *SETBP1* and its targets.

### TF activity

We acquired prior knowledge on the direction of TF regulation from CollecTRI (accessed May 2023) [26] and combined it with GTEx expression TPM to infer TF activity using decoupleR [27] (v.2.6.0). We used a multivariate linear model (run_mlm) with a minimum threshold of 5 targets per TF to calculate activity scores (represented as t-values) for all 758 TFs. We scaled and centered data before summarizing each TF’s average regulator activity. Positive and negative scores denote TF activity and inactivity, respectively.

## Results

**Fig 2:**
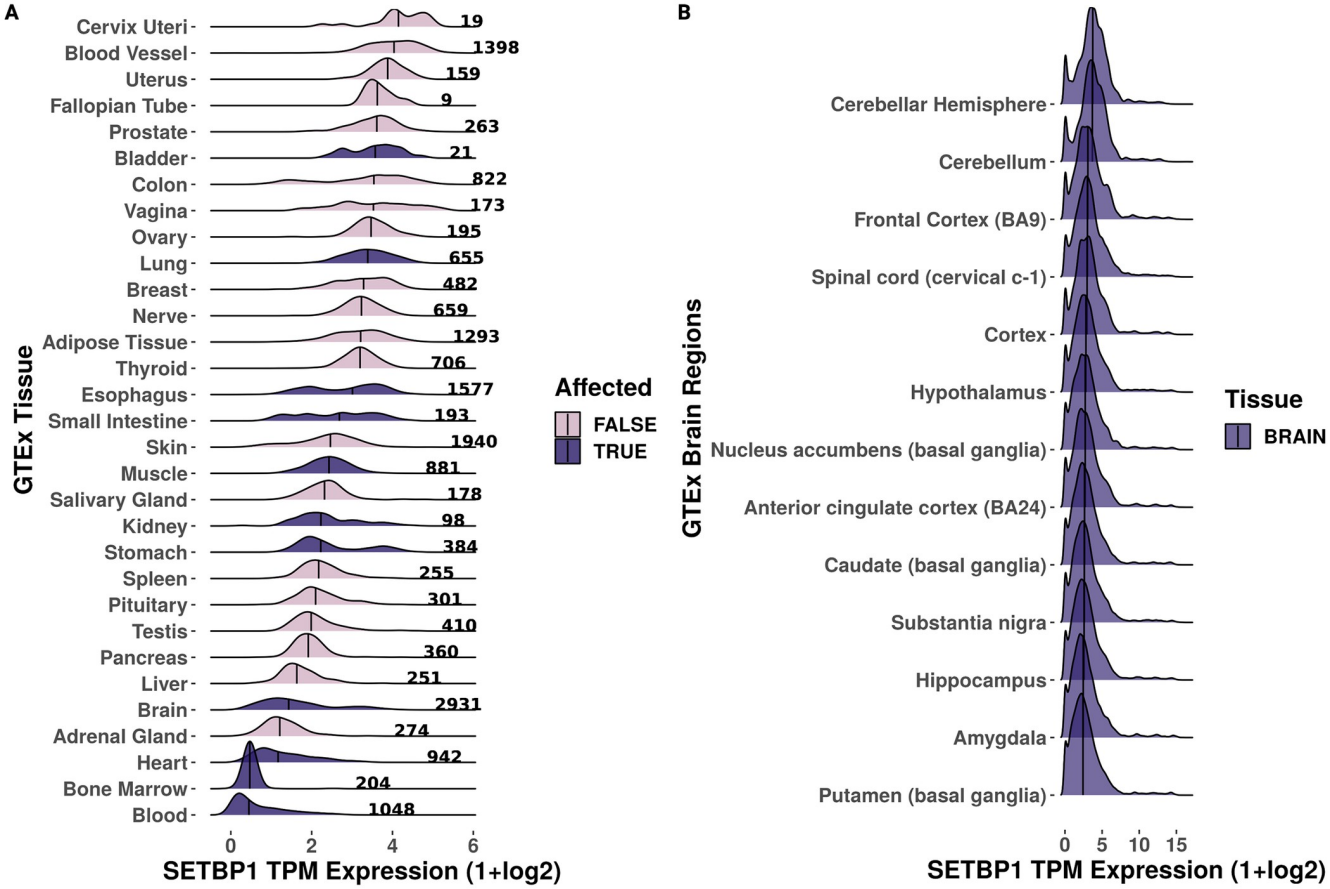
Gene expression of *SETBP1* across GTEx tissues. Ridgeline plot of the scaled median TPM values (x-axis) **(A)** across samples (denoted by bold number) for each GTEx tissue (y-axis) and **(B)** brain subregions (y-axis) for *SETBP1* where disease-affected tissues are colored by dark purple and pink for true and false, respectively. The median is denoted by the vertical black line.

We first examined the gene expression of *SETBP1* across 31 adult human tissues from the GTEx consortium (n = 19,081 samples total). We found that while *SETBP1* was expressed ubiquitously (median TPM range, 0.364–16.719; Fig 2A), it was most highly expressed in the cervix, blood vessel, and uterus (median TPM 16.719, 15.422, and 13.730, respectively) and most lowly expressed in the blood, bone marrow, and the adrenal gland (median TPM 0.135, 0.384, and 0.963, respectively). When we investigated the brain-region-specific expression of *SETBP1*, we found it was similarly expressed across subregions (Fig 2B).

**Fig 3:**
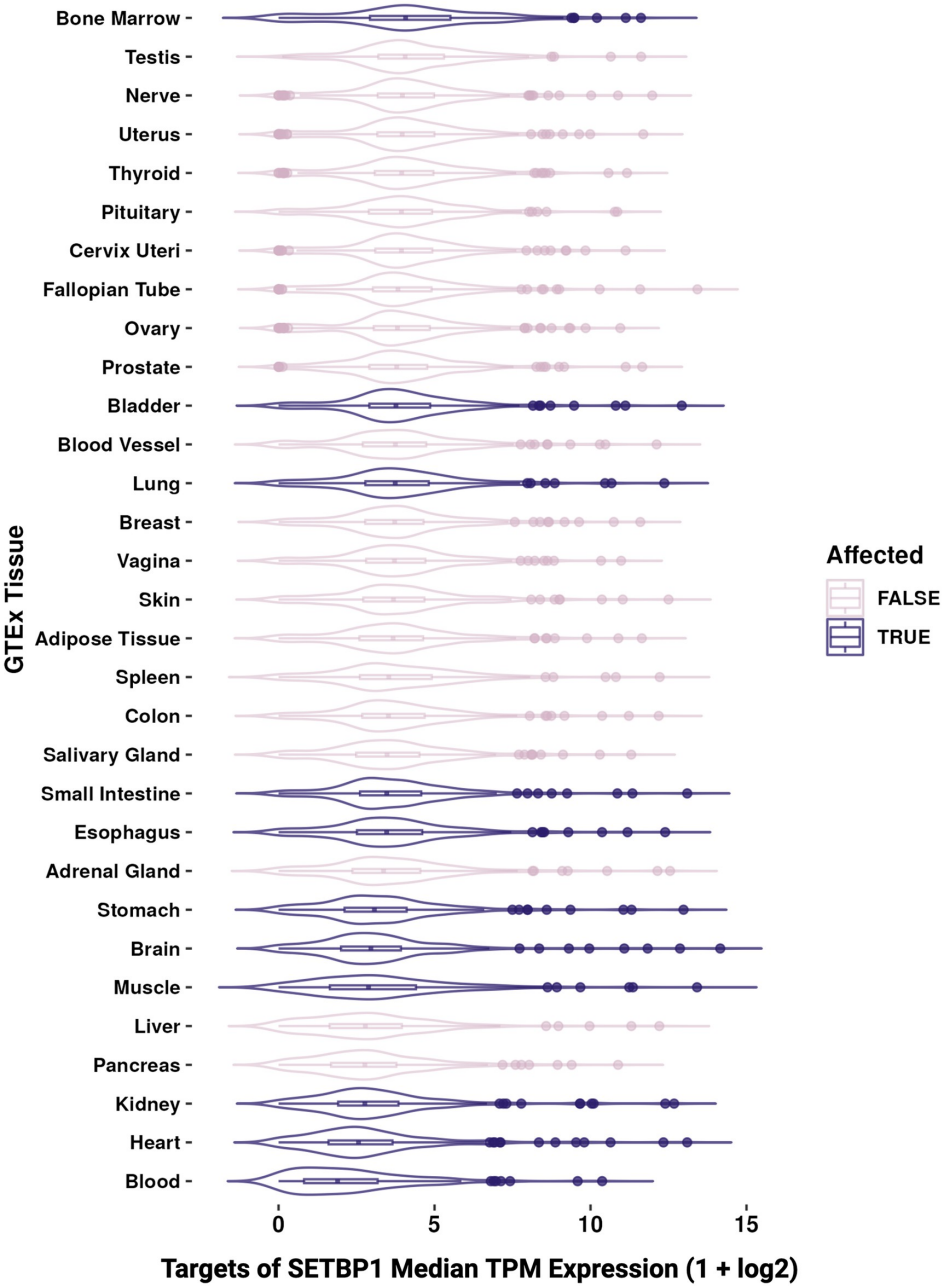
Gene expression of known SETBP1 targets across GTEx tissues. Boxplot of the scaled median TPM values (x-axis) across samples for each GTEx tissue (y-axis) for known targets of SETBP1 where disease-affected tissues are colored by dark purple and pink for true and false, respectively.

Further, we examined the gene expression of 209 known SETBP1 targets we previously compiled [11] from SETBP1 ChIP-seq binding sites [28], MSigDB [29], SIGNOR [30], TRRUST [31], Piazza et al. [2], and Antonyan et al. [10]. We found that most known SETBP1 targets were also broadly expressed across adult human tissues (62.2% of known SETBP1 targets had a TPM>3 in >90% of tissues; Fig 3).

### Targets of SETBP1 are widely expressed across tissues and cluster by gene expression into functionally distinct pathways

**Fig 4:**
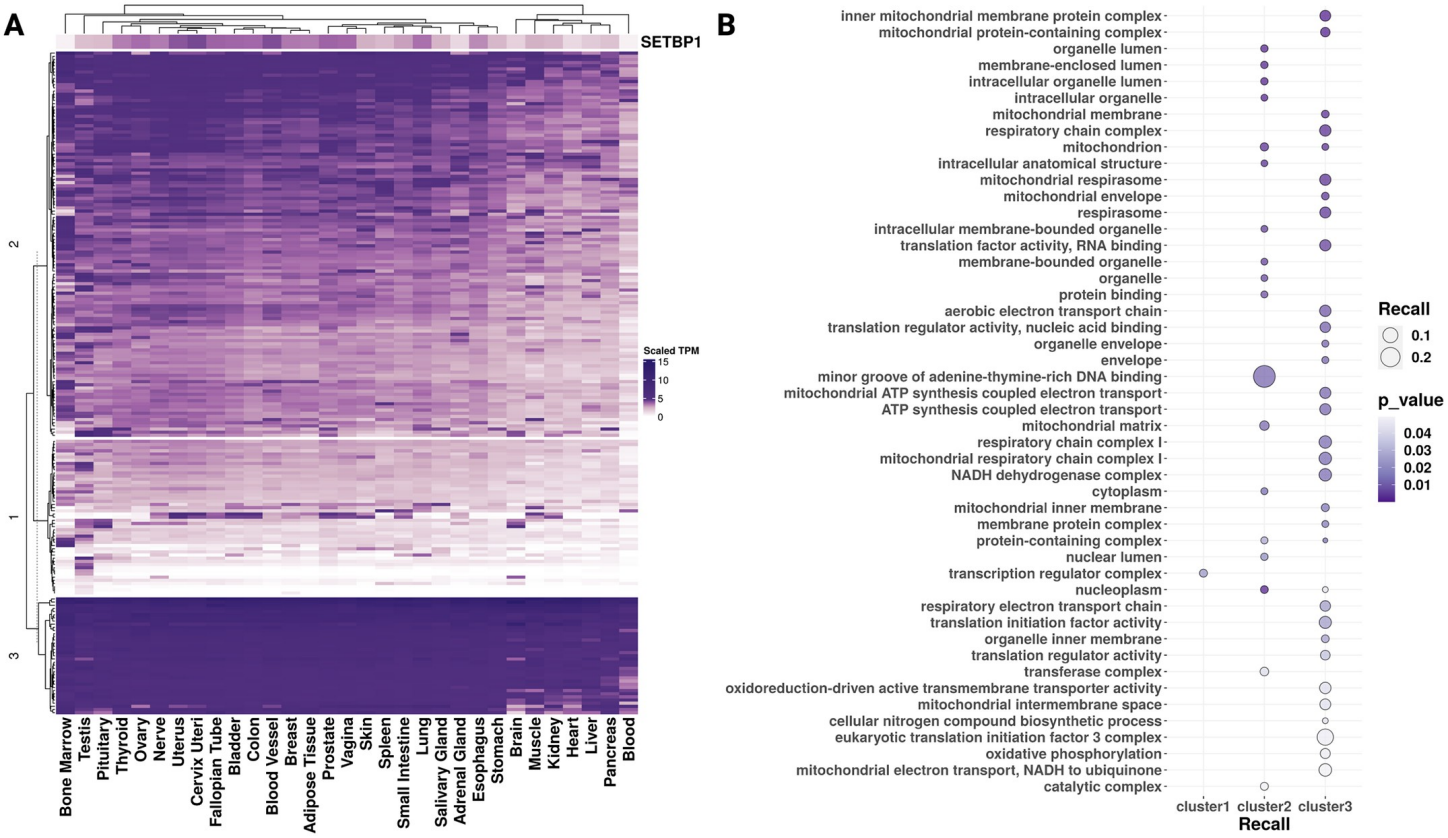
Gene expression of *SETBP1* and its known TF targets across GTEx tissues. **(A)** Heatmap clustering (y-axis) *SETBP1* and its known TF targets’ scaled TPM gene expression by tissue (x-axis). **(B)** Dot plot representing the FEA results for genes from clusters 1, 2, and 3 identified in A. Here dot size indicates recall, the proportion of functionally annotated input genes to each term’s full geneset size (y-axis), and color indicates significance (darker purple; more significant).

We next assessed the variability of SETBP1 targets’ expression across tissues and if particular functions were enriched based on their co-expression across GTEx tissues. When clustering *SETBP1* targets by gene expression, we selected three k-means clusters by both the elbow and Trace(W) method (S1 Fig, S2 Table) and found a higher correlation of expression between *SETBP1* and its targets in clusters 1 and 2 than in cluster 3 (rho ranges -0.25 to 0.82, -0.28 to 0.84, and—0.46 to 0.69, respectively) **(**S2 Table). The targets whose gene expression most correlated to *SETBP1* included *ZEB1* (cluster 2, rho 0.84, p-value = 4.63e-9), *TTC23* (cluster 2, rho 0.82, p-value = 1.14e-8), *RALGAPA1* (cluster 1, rho 0.82, p-value = 1.77e-8), *RAD52* (cluster 2, rho 0.82, p-value = 2.13), *CZIB* (cluster 2, rho 0.81, p-value = 3.07e-8), and *PBRM1* (cluster 2, rho 0.77, p-value = 3.54e-7). The most anti-correlated genes were the mitochondrial-associated genes *MT-RNR1* and *MT-TF* (cluster 3, rho -0.462 and -0.459, p-value = 0.89e-2 and 0.94e-2, respectively). Complete linkage hierarchical clustering of all tissues revealed one clade that included predominantly SETBP1 disease-affected tissues (5 out of 7), including brain, muscle, kidney, heart, and blood (Fig 4A). We further analyzed if the SETBP1 gene targets within these k-means clusters had similar functions using over-representation analysis and found that clusters 1, 2, and 3 included genes enriched for the transcription regulator complex, minor groove AT-rich DNA binding, and mitochondrial structure and function, respectively (Fig 4B).

### SETBP1 TF activity is decreased in blood and increased in the pituitary

**Fig 5:**
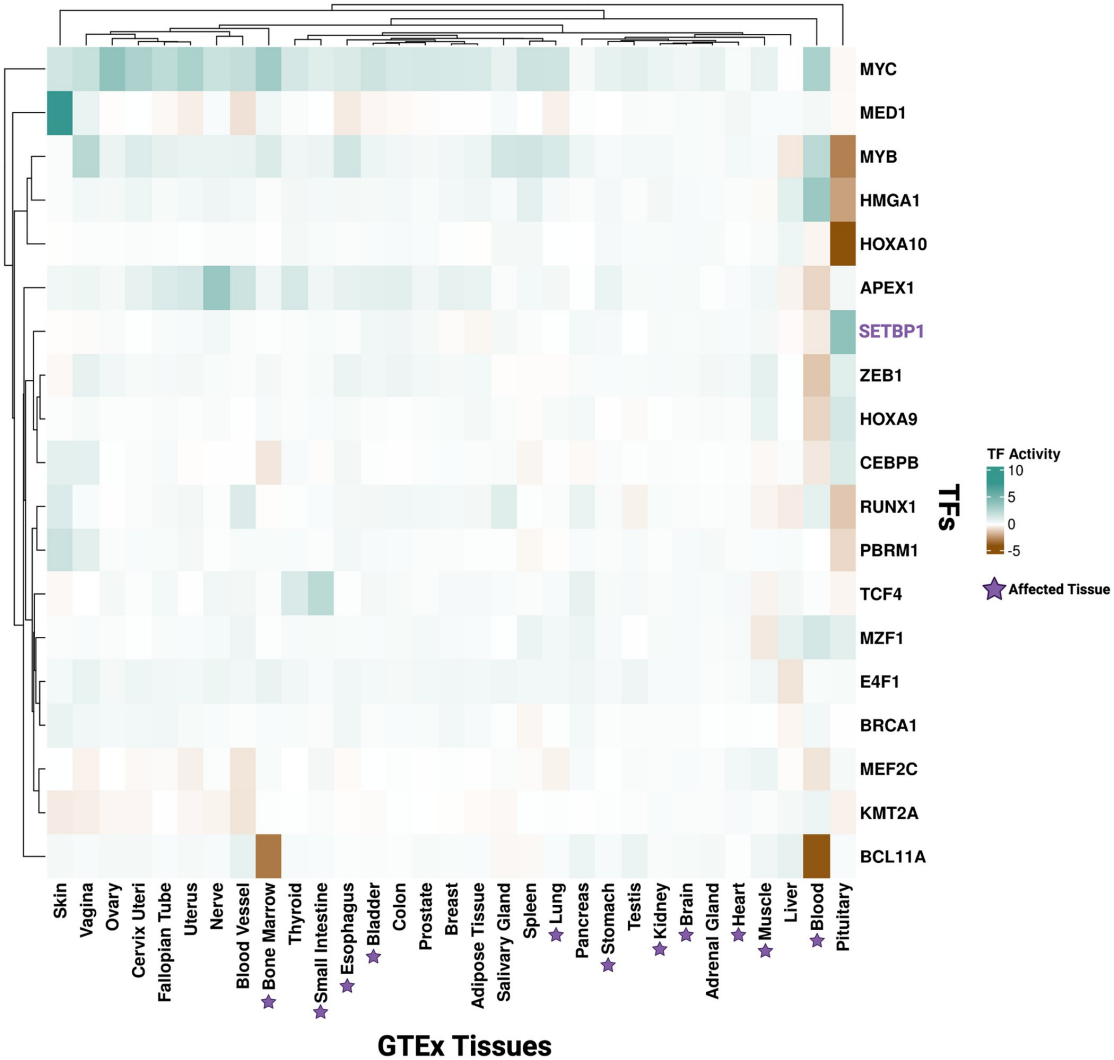
Tissue-specific TF activity of SETBP1 and TFs it targets. Heatmap representing TF activity scores of SETBP1 (purple) and its known TF targets (black, y-axis) across GTEx tissues (x-axis). Affected tissues in SETBP1-associated diseases are indicated by a purple star (x-axis). Teal and brown represent active and inactive TF activity, respectively.

Due to its role as a TF, we next inferred the TF activity of SETBP1 across GTEx tissues. We did this by extracting the direction of SETBP1’s regulation of each target for each tissue based on prior knowledge in CollecTRI, a comprehensive, species-specific, curated database of TFs and their transcriptional targets. Using decoupleR, we then built a multivariate linear model taking into account the direction (repressing or activating) of TF-target interactions weighted by the GTEx gene expression values. For each tissue, we interpreted positive activity scores as indicating a TF, in this case, SETBP1, is active, negative scores as indicating a TF is inactive, and zero as indicating a lack of coordinated regulation by the TF **(**Fig 5). We found that across tissues, SETBP1 TF activity was mostly near zero (S1 Table; range: -0.739 to 3.760 with median = -0.024 and variance = 0.53), except pituitary (TF activity = 3.760 indicating SETBP1 is actively regulating its targets) and blood (TF activity = -0.739 indicating SETBP1 is inactive). Except for blood, we did not find that tissues known to be affected by SETBP1-associated diseases had strong evidence of SETBP1 regulation activity in non-diseased adult tissues.

### SETBP1 TF targets demonstrate tissue-specific TF activity

In addition to SETBP1, we also inferred TF activity for targets of SETBP1 that function as TFs (Fig 5). We found that these TFs, in addition to SETBP1, had a lack of coordinated TF activity in the brain, heart, and kidney, all of which are SETBP1-HD or SGS affected tissues (median = -0.004, variance = 0.008 for brain; median = -0.034, variance = 0.014 for heart; and median = -0.026, variance = 0.022 for kidney). However, SETBP1 targets with previously reported functions involving cancer and DNA damage had variable TF activity in the blood. For example, we predicted BCL11A (linked to multiple blood cancers as reviewed in [32, 33]) is inactive in both blood and bone marrow under non-diseased conditions (TF activity -4.124 and -3.287, respectively). MEF2C (often deregulated and associated with recurrence in leukemia [34]) and ZEB1 (shown to modulate hematopoietic stem cell fates [35, 36]) are also inactive in the blood (TF activity -0.882 and -1.532, respectively).

Furthermore, prior research showed that oncogenic apoptosis resistance and unresolved DNA damage signatures persist due to *SETBP1* variants in SGS [11, 37]. Here, we found that in non-diseased adult tissue, TCF4, a known apoptosis regulator [38, 39], and APEX1, part of the SET complex in DNA damage response [10], also had variable TF activity across SGS-affected tissues. We predicted TCF4 to be inactive in muscle (TF activity = -0.553) and have an active role in the small intestine (TF activity = 2.181) (Fig 5). Additionally, we found APEX1 had a predicted loss of activity in the blood (TF activity = -1.810), but was active in the bladder (TF activity = 0.694) and esophagus (TF activity = 0.590) (Fig 5). Furthermore, we identified a lack of coordinated activity in kidney, blood, bone marrow, esophagus and muscle for TCF4 and a lack of coordinated activity in blood, muscle, and heart for APEX1 (Fig 5). In summary, we have provided a map of SETBP1 and TF targets of SETBP1 activity across non-diseased adult human tissues.

### Interactive web application for TF activity

In order to make our research accessible to the broader scientific community, we developed a Shiny web application with pre-computed TF activity scores that we inferred for 758 TFs for each of the 31 GTEx tissues (https://lasseignelab.shinyapps.io/gtex_tf_activity/). This application allows users to search for TFs and compare their activity across all 31 GTEx tissues.

## Discussion

Our study maps tissue-specific molecular signatures associated with SETBP1 across 31 human non-diseased adult tissues. We found that *SETBP1* and its known targets were widely expressed across those tissues, and FEA revealed gene sets related to transcriptional regulation, DNA binding, and mitochondrial function. Further, we uncovered tissue-specific TF activity through TF activity analysis of SETBP1 and its TF targets, underscoring the role of tissue context-driven regulation that may serve to generate hypotheses regarding the importance of TF activity in disease contexts. We also provide a framework for investigating tissue-specific gene expression and TF activity for other genes, particularly those associated with multiple diseases or multi-syndromic diseases with pathophysiological impacts across multiple tissues. As many genes associated with developmental disorders are also associated with a predisposition or increased risk of cancer (reviewed in [40, 41]), applying this framework to those genes may be fruitful.

With complete-linkage hierarchical clustering of SETBP1 target gene expression by tissue, we discovered one clade largely consisted of affected tissues (5 out of 7 of the tissues in the clade), including brain, kidney, heart, and blood, the most frequently noted tissues impacted by SETBP1 perturbations (Fig 4A) [1, 5, 42, 43]. These results suggest that, in non-diseased tissues, there is a similarity in the expression of SETBP1 targets across multiple tissues known to exhibit a phenotype in SETBP1-associated diseases. We further investigated the trends of expression of SETBP1 targets by k-means clustering and the correlation of each target’s expression to *SETBP1* gene expression (Fig 4A, S2 Table). We compared the total within-cluster sum of squares (“elbow” method) as well as Trace(W) method to select 3 k-means clusters [22, 23]. We highlighted the highest correlated and anti-correlated genes associated with SETBP1’s roles as an active or inactive TF [1]. Of note, some of the most significantly correlated and anti-correlated targets included genes with known critical roles in development (*ZEB1*, p-value = 4.63e-9 [44] and *RALGAPA1*, p-value = 1.77e-8 [45]), with additional involvement in metastasis and therapy resistance for *ZEB1* [44]. Among the non-significant highest correlated and anti-correlated genes were *HMGA1* (p-value = 0.13) and *MYB* (p-value = 0.17). Their non-significance in correlation analyses to SETBP1 across tissues may highlight a potential context dependence or tissue-specific regulatory roles. In addition to having functions in organism development, HMGA1 enhances recovery from double-stranded DNA breaks. When overexpressed, it sensitizes cells to DNA damage and is a driver of malignant tumors [46]. DNA damage has also been noted in previous studies involving animal models and cell lines of SGS [10, 11, 37]. Additionally, SETBP1’s consensus binding site largely overlaps with the AT-hook consensus motif of HMGA1 [2]. These results suggest further research investigating the tissue-specific role HMGA1 may play in DNA damage mechanisms for SETBP1-associated cancers and altered neurodevelopment in SGS and SETBP1-HD may be fruitful. On the other hand, *MYB* expression is known to be critical for myeloid leukemia induced by SETBP1 activation, and its inhibition could be beneficial for treating SETBP1-associated neoplasms [1, 47]. The genes we identified here highlight previously known mechanisms underlying SETBP1-associated disease and provide additional potential targets for future investigation.

To uncover potential functional patterns by expression clusters, we also tested each of our 3 k-means clusters of gene targets for over-representation of GO terms where recall represented the proportion of functionally annotated input genes to each term’s full gene set size (Fig 4B). We found cluster 3 genes were ubiquitously and highly expressed across tissues. These genes were enriched for terms regarding mitochondrial structure and function (Fig 4B) and included many mitochondrially-encoded *SETBP1* targets (*MT-ND6*, *MT-RNR1*, *MT-TE*, *MT-TP*, *MTCO3P12*, *MT-TF*) (S2 Table). Cluster 2 exhibited the highest recall with GO terms enriched for minor groove AT-rich DNA binding (Fig 4B), similar to regulation by SETBP1 as well [2]. Cluster 1 genes enrichment was for just one GO term, transcription regulator complex (Fig 4B), and included genes for the cancer-associated transcription factors *RUNX1*, *HOXA9*, and *MYB* [48]. These results further highlight the varying roles of SETBP1 targets and their expression patterns across tissues.

Studying both gene expression and TF activity data enables the quantification and visualization of how gene expression and TF regulation change across tissues. For example, while SETBP1 shows increased TF activity in the pituitary compared to other tissues, expression of the *SETBP1* gene itself is variable in the pituitary. This analysis emphasizes the tissue-specific difference in the expression and regulation activity of SETBP1. Furthermore, while oncogenesis can drive neoplastic cells to perturb local and systemic homeostasis involving pituitary hormones, there is no known clinical phenotype in the pituitary for *SETBP1*-associated disease. The difference across expression and TF activity could be highlighting a previously undiscovered molecular phenotype involving the pituitary. Out of all SETBP1 disease-associated tissues, blood was the only tissue exhibiting notable SETBP1 TF inactivity. Previous studies support that surpassing a higher functional threshold (i.e., more damaging or impacting variants) is required for SETBP1-driven cancer [43]. Our findings here with respect to SETBP1 in non-diseased blood, suggest that GoF variants (already known to drive blood-associated cancers such as myeloid leukemia [16, 43, 49]) may impact SETBP1’s inactive TF role, suggesting future research directions that may shed light on the mechanism behind SETBP1-associated blood cancers. For example, SETBP1 has been shown to activate *MYC* [50], which we calculated has a TF activity score of 2.77 in the blood and positive TF activity scores in 27 out of 30 other tissues (Fig 5). Likewise, we and others [10, 11, 37] have hypothesized that in the presence of pathogenic germline variants, alterations in TCF4 or APEX1 activity lead to apoptosis resistance or increased DNA damage in SGS. Our results suggest both exhibit active TF roles across many tissues in non-diseased adult tissues, so disease-associated perturbations may impact SETBP1 TF activity, contributing to SGS.

Our study relies on the assumption that TF protein activity can be inferred by the weighted mode of regulation and transcript levels of its target genes [27]. A major limitation of this study is that we conducted analyses in adult bulk expression profiles. For disease-associated genes with developmental phenotypes like *SETBP1*, temporal expression [51, 52] and TF activity are likely key to linking disrupted genes to molecular and physiological phenotypes. Comprehensive prenatal and postnatal gene expression atlases in active development (e.g., developmental GTEx, dGTEx) will provide an unprecedented opportunity to repeat and expand the analyses in this study across developmental time points. Additionally, as we conducted the analyses here in bulk profiles, we cannot assess the gene expression and TF activity of particular cell types. Therefore, further research could utilize single-cell transcriptomics data or cell expression atlases to explore the cell-type-specific expression and functional variation of *SETBP1* across cell types within a tissue. However, as we recently reported, SETBP1’s role as an epigenetic hub leads to cell-type-specific differences in TF activity, gene targeting, and regulatory rewiring in the mouse cerebral cortex and kidney [11]. This underscores the importance of future studies that generate and analyze the necessary data to understand cell-type-specific gene expression and TF activity across human tissues. Furthermore, some affected tissues related to the skeleton and eyes are not included within GTEx v8. If this data becomes available, future studies could investigate tissue-specific expression and activity for vision loss, craniofacial, and skeletal abnormalities within SGS and SETBP1-HD. Finally, the GTEx tissues may have subclinical pathologies that have not been previously reported, so care must be taken with the interpretation of non-affected tissues. To gain a more comprehensive understanding of the expression and functionality of *SETBP1* and its target genes across tissues, future experiments could also leverage cell lines or animal models to investigate the regulatory mechanisms of *SETBP1* and subsequent TF activity.

## Conclusions

In summary, our study highlights the importance of considering tissue-specific expression and regulatory properties in investigating disease-related genes. It provides a basis for future investigations of TFs involved in processes across many tissues, including developmental and cancer contexts.

## Supporting information

S1 Table TF activity across tissues. TF activity scores acros GTEx tissues using prior TF knowledge from CollecTRI and using a mulivariate linear model with decoupleR.
(XLSX)

S2 Table Target clustering and correlations. SETBP1 target genes clustering assignment by GTEx gene expression across tissues and correlation to *SETBP1* gene expression.
(XLSX)

S1 Fig Determining optimal k-means clusters. K-means clustering indices of GTEx scaled normalized *SETBP1* and gene targets’ expression using (**A**) Elbow plot, 1–15 k-means clusters (x-axis) plotted by their total within-cluster sum of squared distances (inertia), where dashed blue line signifies the point at which the inertia decreases and represents a sufficient number of clusters. (**B**) Line plot of Trace(W), the sum of the diagonal of the sum of squared within-group dispersion matrix (y-axis) for each cluster (x-axis) is used to calculate second differences, and the optimal cluster (dashed blue line) is indicated as the maximum value between levels.
(TIFF)

## Abbreviations

FEA: Functional Enrichment Analysis
GoF: gain of function
GTEx: Genotype-tissue expression
LoF: loss of function
OMIM: Online Mendelian Inheritance of Man
RNA-seq: RNA-sequencing
SETBP1-HD: SETBP1 haploinsufficiency disorder
SGS: Schinzel Giedion Syndrome
TF: Transcription Factor
TPM: Transcripts per Million
