# Supplementary figures and images for: The landscape of SETBP1 gene expression and transcription factor activity across human tissues
Source: PLoS One. 2024 Jan 2;19(1):e0296328. doi: 10.1371/journal.pone.0296328 (PMC10760659; doi:10.1371/journal.pone.0296328)

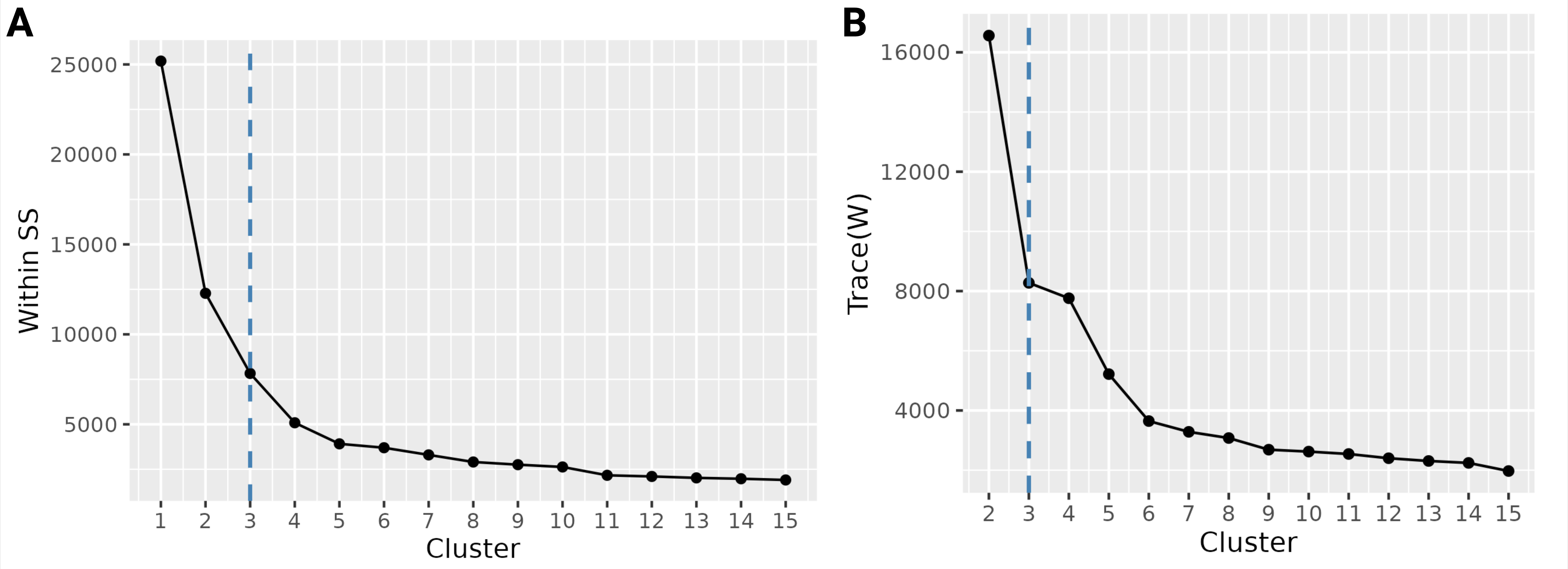

Supplement: S1 Fig — K-means clustering indices of GTEx scaled normalized SETBP1 and gene targets’ expression using (A) Elbow plot, 1–15 k-means clusters (x-axis) plotted by their total within-cluster sum of squared distances (inertia), where dashed blue line signifies the point at which the inertia decreases and represents a sufficient number of clusters. (B) Line plot of Trace(W), the sum of the diagonal of the sum of squared within-group dispersion matrix (y-axis) for each cluster (x-axis) is used to calculate second differences, and the optimal cluster (dashed blue line) is indicated as the maximum value between levels. (TIFF) [file pone.0296328.s003.tiff]
